# How Our School Makes Doctors

**Published:** 1880-11

**Authors:** Josephus Eleanorus Kinslow

**Affiliations:** Professor of Diseases of the Umbilicus in the Dreamtown Medical College


### HOW OUR SCHOOL MAKES DOCTORS.

BY JOSEPHUS ELEANORUS KINSLOW, M. D., PROFESSOR OF DISEASES OF THE UMBILICUS IN THE DREAMTOWN MEDICAL COLLEGE.

Since my last writing, I have started a medical college in Dreamtown, and am running it in conjunction with three other professors. For the benefit of other schools I will give you a description of our college.

Medical schools being so very scarce, and traveling expenses so exorbitant, the legislature of Hoosierland thought a school indispensable to Dreamtown and therefore furnished us a perpetual charter.

"Dreamtown is a flourishing little town on the bank of the Vandyke creek," so says our catalogue (which, by the way, has a flashing red cover and therefore fills a long felt want; every doctor in the land should have it), "having five hundred inhabitants, several groceries where powder and coal oil are sold, and the farmers who live in our township run threshing machines, etc. This, as any medical student can see, furnishes our clinics with occasional material. Boarding at $1 50 per week; students clubbing together can get boarding for nothing, the whole course need not cost more than $75 (whisky included.)"

"The Faculty," so continues the catalogue, "consists of four members: a professor of anatomy, physiology, materia medica, chemistry and forensic medicine; a professor of surgery, obstetrics, therapeutics and applied medicine; a professor of specialties; and a professor of the diseases of the umbilicus, who (the professor) also runs the college, being dean, secretary, treasurer, and door keeper. The text-books are a specialty: Practice, Ziemssen's Cyclopædia; Surgery, Holmes' System; of Obstetrics, Obstetrical Plates; Umbilicus, Kinslow's Cyclopedia of."

Two sessions, each of four months (including six weeks vacation), are given every year and students that enter our college (if students cannot write their name, so much the better) will get a guarantee that they will receive a sheep-skin at the end of eight months.

Four lectures will be delivered daily and one political speech will be delivered every Saturday afternoon in the college. The especial thing we wish to hold forth is that we own a microscope (bought it second hand for $15), an electrical machine (cost $12), an amputating case, etc.

The fees of our college are cheap, seeing that times are hard: General fee, $30; graduation fee, $5. An occasional drummer is killed (always the same one) and dissected; but we charge no fee for this. Our college edifice is not surpassed by any in the country, it consists of a frame house 20 by 40 feet, having one room (lecture room), a cellar, (laboratory), and a garret (the drummer is dissected here).

We do not belong to the A. M. C. A., and will not apply for admission, as we consider that august assembly a fraud, because it wants to get up "three terms," "higher fees," "better medical education," etc.; such stuft will break up our college. All the graduates of our school are furnished with instructions how to gain practice, and those of our graduates who wish to get two diplomas, we recommend to a certain hospital school in the West, which, at least, did once promise to furnish its graduates with practice. Our premiums are three chromos, given to the three best students. We will soon give out a semi-centennial medical journal, "The Medical Insinuator," in connection with our school.

I hope that this writing will induce every young man and every young woman (who is out of employment) to send for a catalogue and to pitch in We make no difference of sex in our students, taking boys and girls, men and women, promiscuously. The age for graduation is twenty-one (more or less); as to having a good moral character, etc., we care not. DREAMTOWN HOOSIERLAND.

